# Comparison of two doses of leucovorin in severe low-dose methotrexate toxicity – a randomized controlled trial

**DOI:** 10.1186/s13075-023-03054-2

**Published:** 2023-05-19

**Authors:** Mudit Bhargava, Chirag Rajkumar Kopp, Shankar Naidu, Deba Prasad Dhibar, Atul Saroch, Alka Khadwal, Tarun Narang, Siddharth Jain, Aastha Khullar, Bidya Leishangthem, Aman Sharma, Susheel Kumar, Shefali Sharma, Sanjay Jain, Varun Dhir

**Affiliations:** 1 Division of Rheumatology, Department of Internal Medicine, Postgraduate Institute of Medical Education and Research, Chandigarh, 160012 India; 2 Division of Emergency Medicine, Department of Internal Medicine Postgraduate Institute of Medical Education and Research, Chandigarh, 160012 India; 3 Department of Clinical Hematology and Medical Oncology, Postgraduate Institute of Medical Education and Research, Chandigarh, 160012 India; 4 Department of Dermatology, Postgraduate Institute of Medical Education and Research, Chandigarh, 160012 India

**Keywords:** Methotrexate, Toxicity, Folinic acid, Leucovorin, Overdose, Poisoning, Adverse drug reaction, Cytopenia, Neutropenia, Randomized controlled trial

## Abstract

**Background:**

Leucovorin (folinic acid) is a commonly used antidote for severe toxicity with low-dose methotrexate, but its optimum dose is unclear, varying from 15 to 25 mg every 6-h.

**Methods:**

Open-label RCT included patients with severe low-dose (≤ 50 mg/week) methotrexate toxicity defined as WBC ≤ 2 × 10^9/L or platelet ≤ 50 × 10^9/L and randomized them to receive either usual (15 mg) or high-dose (25 mg) intravenous leucovorin given every 6-h. Primary outcome was mortality at 30-days and secondary outcomes were hematological recovery and mucositis recovery. Trial Registration number: CTRI/2019/09/021152.

**Results:**

Thirty-eight patients were included, most with underlying RA who had inadvertently overdosed MTX (taken daily instead of weekly). At randomization, the median white blood and platelet count were 0.8 × 10^9/L and 23.5 × 10^9/L. 19 patients each were randomized to receive either usual or high-dose leucovorin. Number (%) of deaths over 30-days was 8 (42) and 9 (47) in usual and high-dose leucovorin groups (Odds ratio 1.2, 95% CI 0.3 to 4.5, *p* = 0.74). On Kaplan–Meier, there was no significant difference in survival between the groups (hazard ratio 1.1, 95% CI 0.4 to 2.9, *p* = 0.84). On multivariable cox-regression, serum albumin was the only predictor of survival (hazard ratio 0.3, 95% CI 0.1 to 0.9, *p* = 0.02). There was no significant difference in hematological or mucositis recovery between the two groups.

**Conclusion:**

There was no significant difference in survival or time-to hematological recovery between the two doses of leucovorin. Severe low-dose methotrexate toxicity carried a significant mortality.

**Supplementary Information:**

The online version contains supplementary material available at 10.1186/s13075-023-03054-2.

## Key messages

1. What is already known about this area?
  We searched the PUBMED using the terms "Methotrexate"[Mesh] AND “toxicity” and could not find any randomized controlled study which compared different doses of leucovorin in low-dose methotrexate toxicity. Leucovorin is generally used at a dose of 10 to 25 mg every 6hourly (varies by center) in low-dose methotrexate toxicity – extrapolated from regimens followed for high-dose MTX rescue.
2. What new does this study add?
  - First RCT to compare two different doses of leucovorin in severe-low dose methotrexate toxicity
  - There was no significant difference in mortality or time-to-hematologic recovery or mucositis recovery between the doses of 15 or 25 mg every 6-h in low-dose methotrexate toxicity. There was substantial mortality in both groups ranging from 42-47% despite leucovorin, with the common cause of death being sepsis.
3. How might this impact clinical practice or future development?
  - Higher doses of leucovorin than 15 mg every 6 h unlikely to help to improve mortality, rather better critical care may help. Unclear whether lower doses may be as beneficial.
  - Need to emphasize preventive aspect of MTX toxicity– physicians should emphasize the unique dosing of MTX to patients and give a warning against daily use at the time of consultation.

## Introduction

Methotrexate (MTX) was introduced in the late 1950s and quickly replaced its congener aminopterin as the preferred antifolate drug [1]. Its uses can be classified into three major categories—high dose (HD-MTX, more than 500 mg) used for malignancies, medium dose for gestational trophoblastic diseases and low-dose (LD-MTX, up to 50 mg per week) for its anti-inflammatory/immunomodulatory property in rheumatological and dermatological diseases [2].

Apart from superior efficacy and long-term continuation rates, LD-MTX also has an excellent safety profile, and these features have led to its widespread use in rheumatology for a variety of indications, most common being rheumatoid arthritis [3]. However, severe toxicity can arise with inappropriate use, like inadvertent daily intake or administration in renal failure. The consequent toxicity which arises can be severe and be associated with high mortality rates [4].

Leucovorin or folinic acid (initially called citrovorum factor) was discovered in 1948 as a growth factor for the bacterium *Leuconostoc Citrovorum* [5]. Its property of reversing the folate block by antifolate drugs led to its being launched as a drug (calcium salt) for antifolate toxicity in 1957 (Lederle, American Cyanamid Co) [6]. Currently, it is primarily used as rescue therapy (prevent severe toxicity) with HD-MTX infusion, at a dose of 10 to 15 mg per m^2^ IV every 6 h till MTX levels are below 0.1 μM. However, depending on the serum MTX levels, its dose can increase to 150 mg q3hourly [7]. In LD-MTX toxicity, monitoring serum MTX levels has no established role and the dose of leucovorin used is empirical varying from 10–25 mg q6 hourly.

At our tertiary care referral center, we had been treating severe LD-MTX toxicity with leucovorin at a dose of 15 mg every 6 h but found significantly high mortality (unpublished). We speculated that a higher dose of leucovorin may lead to faster hematological recovery and consequently improve outcomes. Thus, we planned this study to compare two different doses of leucovorin, usual (15 mg) or high-dose (25 mg), given intravenously every 6-h.

## Methods

### Study design and participants

FLIMT (Folinic acid/Leucovorin In Methotrexate Toxicity) was a single-center, open-label, pragmatic randomized controlled trial that compared two doses of intravenous leucovorin in severe low-dose methotrexate toxicity. It was conducted from 9 February 2019 to 1 January 2022 in a single-center tertiary-care teaching hospital in North India. The study was approved by the Institutional Ethics Committee of Postgraduate Institute of Medical Education and Research. A written consent was taken from the patients included in the study. The trial was prospectively registered at the Clinical trial registry of India, a primary Register of the International Clinical Trials Registry Platform (ICTRP). Registration number: CTRI/2019/09/021152.

Patients being treated with low-dose methotrexate (≤ 50 mg per week) for any indication who were between the age of 12 to 90 years and had developed severe methotrexate toxicity due to either inadvertent overdose or renal failure (or unknown cause), were eligible to be recruited into the study. Severe toxicity was defined as presence of at least one severe cytopenia (either WBC ≤ 2 × 10^9/L or platelet ≤ 50 × 10^9/L). At enrolment, the patients must not have received more than four leucovorin doses. It was planned to recruit 60 patients, but due to slow recruitment, it was terminated when 38 participants had been recruited.

### Intervention and procedures

Patients were randomly assigned in 1:1 ratio to receive intravenous leucovorin every 6 h at either a usual (15 mg) or high-dose (25 mg), continued till hematologic recovery or for 10-days whichever was earlier. They continued to receive standard care by their treating physician, which included antibiotics, G-CSF, blood products, local analgesics for mucositis. All patients underwent daily blood counts and examination for mucositis for progression/recovery. If discharged from the hospital, patients were telephonically called up at 30-days to assess their outcome.

### Outcomes

The primary outcome (*unplanned*, *initially secondary outcome*) was mortality at 30 days after randomization. Secondary outcomes were time-to-hematological recovery, defined as WBC ≥ 4 × 10^9/L and platelet count ≥ 100 × 10^9/L *(initially was primary outcome)* and time-to-recovery of oral mucositis (to grade 1 or 0 as defined on WHO-mucositis scale). In addition, time-to-recovery of diarrhea was also kept as a secondary outcome but due to incomplete data was not considered in the final analysis.

### Randomization and blinding

Patients were randomized by an online random number generator using permuted blocks of variable size (4, 6 and 8) into two groups by a physician not involved in the study. Allocation was concealed using sequentially numbered opaque sealed envelopes. After recruitment of the patient, the dose of leucovorin was known to the attending nurse, treating physician, trial physician and the patient.

### Statistical analysis

The sample size calculated with presumed recovery to be 7 days and 4 days in the usual and higher dose leucovorin acid (leucovorin) with SD 3, power 95% and alpha error of 0.05, allocation ratio of 1:1, comes to 29 in each group (total 58, rounded to 60) (Program used G power 3.2). All outcomes were assessed using intention-to-treat analyses, and all patients who had been randomized to a particular group were analysed. Baseline continuous variables were compared using t-test for independent samples or Mann–Whitney U test (based on assessment of normality of data) and categorical vaiables using the chi-square test.

The primary outcome was analyzed used chi-square statistics and odds ratio was calculated. In addition, we also performed an adjustment for significant differences at baseline done using logistic regression. Survival analyses were performed using Kaplan–Meier test and log-rank test was used to compare survival between treatment groups. Cox-regression was used to generate hazard ratios and adjust key outcomes for baseline differences. It was also used to assess factors associated with survival—initially univariable analysis were done, and all independent variables with *p* < 0.15 were included in the multivariable analysis. All the statistical tests performed were two-sided with significance level of 0.05. Statistical programs used were IBM SPSS statistics version 27, Open Epi toolkit (http://www.openepi.com) and Graph Pad Prism Version 9.

## Results

### Presentation of patients and baseline characteristics

This study enrolled 38 patients with methotrexate toxicity. There was a female preponderance (63%) and most were receiving methotrexate for rheumatoid arthritis. The most common cause of toxicity was erroneous dosing (taken either daily or alternate day instead of weekly) (68%), followed by renal failure (Table 1). Common symptoms at presentation were oral ulcers (35, 92%), nausea (and vomiting) (29, 76%), diarrhea (23, 61%), bleeding (purpura or other sites) (22, 58%) and fever (21, 55%), with a median (IQR) duration of symptoms of 6-days (IQR 4 − 9). On examination, patients had severe mucositis, purpuric rashes, ulceration of psoriatic plaques (Supplementary file, Patient pictures 1–4). Median white blood count and platelet count at presentation was 0.8 × 10^9/L and 23.5 × 10^9/L.

**Table 1:** Baseline characteristics of patients at randomization

	**All patients** ***n* = 38**	**Leucovorin 15 mg, *n* = 19**	**Leucovorin 25 mg, *n* = 19**	***p*-value**
Age, years, mean ± SD	53.6 ± 14.2	51.4 ± 16.7	55.7 ± 11.2	0.4
Gender, female: male	24:14	12:7	12:7	1.0
*Underlying Disease, n (%)*				
Rheumatoid arthritis	25 (66)	11 (58)	14 (74)	0.21
Psoriasis vulgaris	8 (21)	4 (21)	4 (21)	
Other diseases	5 (13)	4 (21)	1 (5)	
Duration of disease^a^, years	6.5 (4–10)	6.5 (2.5–10)	6.5 (4.3–11.5)	0.71
Comorbid HT or DM, n (%)	11 (29)	6 (32)	5 (26)	0.72
*Cause of Toxicity*				
Erroneous overdose^b^	26 (68)	13 (68)	13 (68)	1.0
Cumulative dose of MTX taken erroneously, mg^d^	75 (50–100)	50 (45–87.5)	95 (69–126)	0.04
Pre-existing renal failure, n (%)^c^	14 (37)	7 (37)	7 (37)	1.0
Unknown cause of toxicity, n (%)	5 (13)	2 (11)	3 (16)	0.9
*Baseline investigations*				
Hemoglobin, g/dl	8.4 (7.3–9.3)	8.4 (7.3–9.1)	8.7 (7.2–9.5)	0.73
White blood count (× 10^9/L)	0.8 (0.4–1.8)	0.8 (0.3–1.8)	0.8 (0.4–1.8)	0.66
Platelet count (× 10^9/L)	23.5 (9–77.3)	17 (9–56)	45 (9–78)	0.49
Serum Creatinine, mg/dl	1.5 (0.9–2.4)	1.6 (0.9–2.2)	1.4 (1–2.7)	0.89
AST, IU/L	33 (18–66)	28 (12–52)	37 (30–89)	0.04
ALT, IU/L	41 (17–83)	28 (16–69)	46 (18–86)	0.45
Serum albumin, g/dl	2.7 (2.2–3.2)	2.5 (2.1–3.1)	3.1 (2.5–3.5)	0.045

All values are in median (IQR) unless specified

*Abbreviations AST* Aspartate aminotransferase, *ALT* Alanine aminotransferase, *HT* Hypertension, *DM* Diabetes mellitus, *MTX* Methotrexate

*Normal ranges:* white blood count (× 10^9/L) is 4–11, platelet (× 10^9/L) is 150–450, serum creatinine 0.5–1.3 mg/dl, AST and ALT is ≤ 40 IU/L, serum albumin is 3.5–5.5 g/dl

^a^For underlying disease of rheumatoid arthritis or psoriasis

^b^Methotrexate taken daily or on alternate days

^c^7 patients had both renal failure and overdose (3 in 15 mg group and 4 in 25 mg group)

^d^The cumulative dose of methotrexate was available in 25 patients

Patients were randomized into two groups of 19 patients each. One group received usual (15 mg) and the other high-dose (25 mg) leucovorin every 6-h. There was no significant difference in baseline characteristics except significantly higher serum albumin (mean difference of 0.4 g per dl, 95% CI 0.03–0.85) and cumulative dose of methotrexate (mean difference of 32 mg, 95% CI 5–58) in patients in the 25-mg leucovorin group (Table 1).

### Flow of patients in the study

**Fig. 1:**
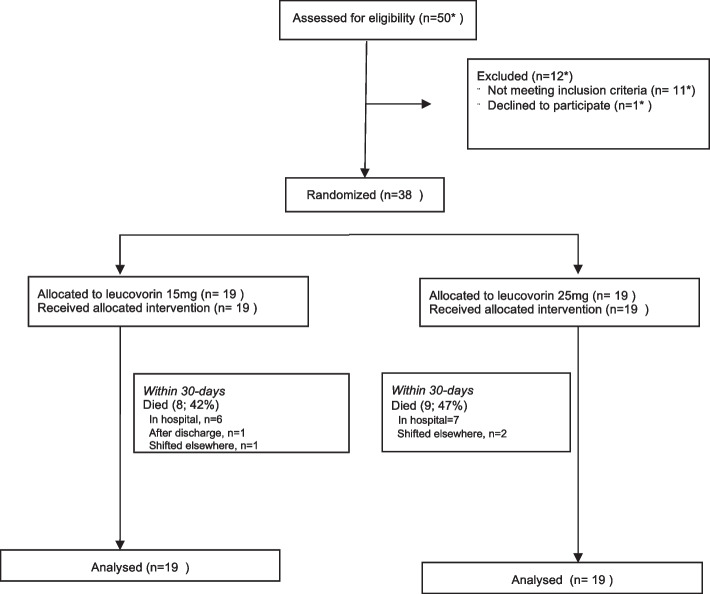
Flow chart showing the flow of patients in the study

All patients received the intervention and were included in analysis. Except 2 patients, all patients received intravenous antibiotics (92%). G-CSF was also given to almost all patients (34/36, 90%). Among 17 patients who died, three patients were shifted to another facility/homecare where they died, whereas one patient died after discharge (Fig. 1).

### Primary outcome (unplanned)

**Fig. 2:**
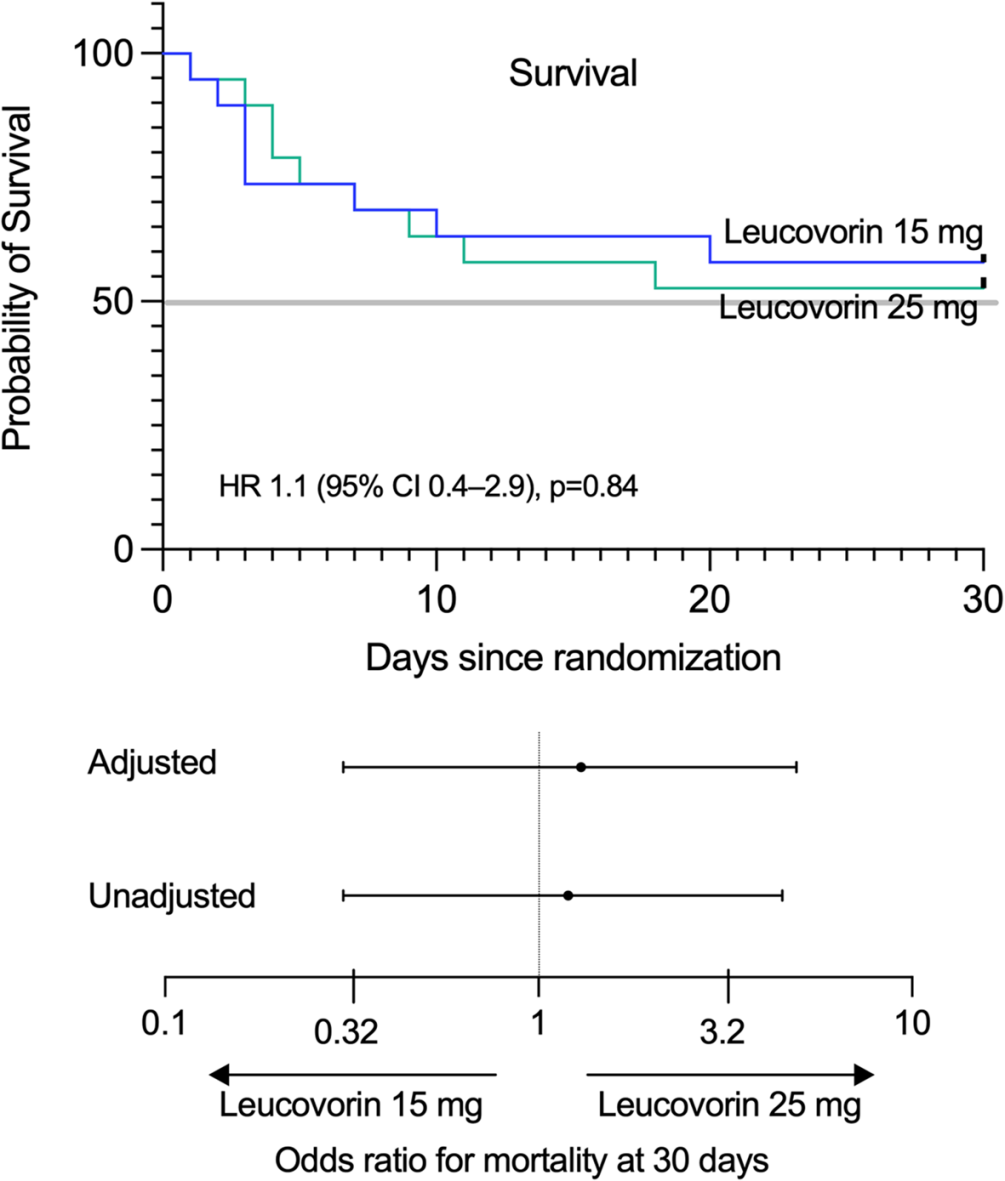
Kaplan–Meier curves showing survival over 30 days in both groups after randomization. Lower panel: Odds ratio for mortality at 30 days between the two dosing strategies

Seventeen patients died over a 30-day period, with no significant difference in the mortality between the 15 (42%) and 25 mg leucovorin groups (47%), *p* = 0.74, inter-group difference (95% CI) -5% (-37 to 26). There was also no significant difference in survival by Kaplan–Meier analysis between the two groups (Hazard ratio of 1.1 (95% CI 0.4–2.9), *p* = 0.84). Even after adjusting for the baseline serum creatinine, there was no significant difference in mortality at 30-days or survival (Table 2, Fig. 2). We also performed an analysis to adjust additionally for cumulative methotrexate dose (available in 25 patients); still there was no significant difference in mortality at 30-days (Odds ratio, 2.2, 95% CI 0.2–21, *p* = 0.5).

**Table 2:** Primary and secondary outcomes

	**Leucovorin 15 mg (*n* = 19)**	**Leucovorin 25 mg (*n* = 19)**	***p*-value**^**#**^	**Unadjusted odds (hazard) ratio**^a^	**Adjusted odds (hazard) ratio**^b^
*Primary Outcome*					
Death by 30 days	8	9			
Mortality % (95% CI)	42 (23–64)	47 (27–68)	0.74	1.2 (0.3–4.5)	1.3 (0.3–4.9)
*Secondary outcomes*					
Time to Hematological recovery, days, median (95% CI)	7 (4–10)	6 (4–8)	0.90	1 (0.4–2.7)	0.8 (0.3–2.1)
Time to Oral ulcer recovery, days, median (95% CI)	5 (2–8)	4 (3–5)	0.59	1.5 (0.6–3.7)	1.4 (0.6–3.5)

*Abbreviations: CI* Confidence interval

^#^*p*-value obtained from univariable analysis by Chi-square for primary and log-rank test for secondary outcome

^a^Comparing 25 mg group to 15 mg group

^b^Adjusted for baseline serum creatinine, Values depict Odds-ratio for the primary outcome and hazard-ratio for the secondary outcomes

Most patients died of septic shock and/or respiratory failure, and one patient died of COVID associated respiratory failure. Most had persistence of severe cytopenia at the time of death (13 (76%)), with recovery in only a few (Supplementary table S1).

### Secondary outcomes

**Fig. 3:**
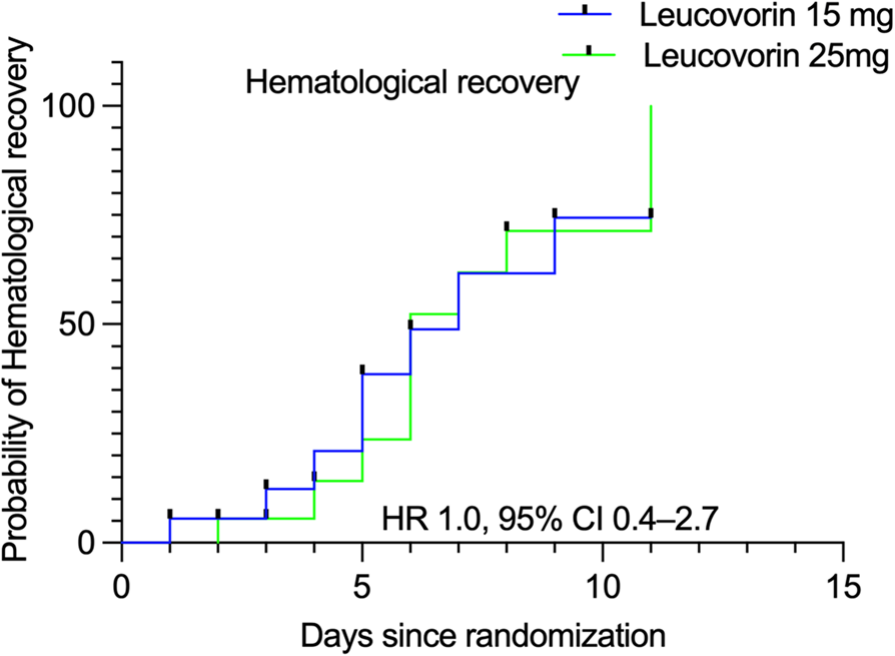
Kaplan–Meier survival curves showing cumulative time-to-event for hematological recovery in the two groups. (Hematological recovery defined as both WBC (≥ 4 × 10^9/L) and platelet recovery (≥ 100 × 10^9/L))

There was no significant difference in the median (95% CI) time-to- hematological recovery (WBC ≥ 4 × 10^9/L and Platelet ≥ 100 × 10^9/L) in the 15 (7 (4.3–9.7)) and 25 mg groups (6 (4.4–7.6) days (*p* = 0.9, hazard ratio 1.0, 95% CI 0.4–2.7) (Table 2, Fig. 3). There was no significant difference in the median time-to-recovery in oral mucositis between the two groups (5, 4 days, *p* = 0.59, hazard ratio 1.5 (95% CI 0.6–3.7) (Supplementary figure S1).

### Other outcomes

There was no significant difference in WBC recovery (WBC ≥ 4 × 10^9/L) platelet partial recovery (Platelet ≥ 50 × 10^9/L), or both WBC and partial platelet recovery between the groups (Supplementary figure S2-S4). Serum methotrexate levels (not available in our institute) were outsourced in two patients and in both these cases they were undetectable In addition, RBC methotrexate polyglutamate 1–3 levels were determined in twelve patients and were within range of those normally found in rheumatoid arthritis patients on methotrexate treatment (Supplementary figure S5).

### Baseline factors associated with death

On univariable and multivariable analysis to assess baseline factors which were significantly associated with survival (cox-regression), only lower serum albumin was found to be significant (hazard ratio 0.2 (95% CI 0.1–0.7) (Table 3). On ROC analysis, serum albumin ≥ 3.1 g/dl at baseline had modest sensitivity and specificity of 57 and 81% for predicting survival (AUC 0.75, *p* = 0.01) (Supplementary table S2).

**Table 3:** Hazard ratios (95% CI) of independent variables which are associated with death in patients with MTX toxicity

	**Cox- Hazard ratio (95% CI)**			
	Univariate		Multivariate	
	Hazard ratio (95% CI)	*P*-value	Hazard ratio (95% CI)	*P*-value
Leucovorin dose^a^	1.1 (0.4–2.9)	0.84		
Age	1.0 (1.0–1.1)	0.97		
Gender (male by female)	1.3 (0.5–3.3)	0.63		
Duration of symptoms	0.9 (0.8–1.1)	0.43		
Comorbidity (HT or DM)	0.5 (0.1–1.5)	0.45		
Dose cumulative MTX	1.0 (0.98–1.0)	0.84		
Hemoglobin at baseline	0.9 (0.7–1.2)	0.52		
WBC nadir (× 10^9)	0.4 (0.2–1.0)	0.06	0.7(0.3–1.7)	0.39
WBC at baseline (× 10^9)	0.6 (0.3–1.1)	0.11		
Platelet count at baseline (× 10^9)	1.0 (1.0–1.0)	0.97		
Platelet count nadir (× 10^9)	1.0 (1.0–1.0)	0.29		
Serum albumin at baseline	0.2 (0.1–0.7)	0.01*	0.3 (0.1–0.9)	0.03*
Creatinine at baseline	1.2 (1.0–1.5)	0.06	1.2 (0.9–1.5)	0.23
Bilirubin at baseline	1.1 (0.7 -1.7)	0.64		

*Abbreviations: MTX* Methotrexate, *WBC* White blood count, *WBC nadir* Lowest WBC count after randomization

^a^25 mg vs 15 mg

^*^p < 0.05

## Discussion

This pragmatic open-label RCT compared two doses of IV leucovorin, 15 and 25 mg given 6 hourly, in patients with severe methotrexate toxicity who were on low-dose MTX therapy, but failed to detect any significant difference in mortality at 30-days or time-to-hematologic recovery between the groups.

A major finding has been the high mortality rate of 42–47% at 30-days in the two groups. On review of previous literature, all of which are retrospective series, mortality rates were reported to be 10–33% [4, 8–10]. A summative review of 70-cases in literature also reported a (lower) mortality of 17% [11]. Thus, it appears that the mortality rate in the present study is much higher than previously reported.

However, a critical analysis finds that previous studies also included patients with mild MTX toxicity, categorized as mild leucopenia or thrombocytopenia. If one closely examines the mortality for patients with severe MTX toxicity, as defined in this study, they were as high or higher than the current study. Scott et al. reported a mortality of 50% among 10 patients with severe toxicity (WBC ≤ 2.0, Hb ≤ 10 g/dl and platelet count ≤ 50 × 109/l) from the United Kingdom [8]. Another series from India reported a mortality of 75% in 16 patients with severe toxicity [9]. The severity of toxicity (by severity of leucopenia) was negatively associated with survival in one study [9]. The current study enrolled patients with severe leucopenia having a median WBC of 0.8 × 10^9/L, and this probably explains the high0-mortality rates.

Most patients died due to severe sepsis and respiratory failure, complicated by ongoing cytopenia. Faster recovery of cytopenia could be reasonably expected to improve mortality by possibly controlling sepsis. In the current study, hematological recovery took a median of 6–7 days, with no significant difference between the two doses of leucovorin. This lag-time (and the failure of higher dose to shorten it) is probably related to rescue being possible only of progenitors on the ‘brink’ but not those that are fatally or irreversibly damaged. A previous report showed severe aplasia in bone marrow aspirates from patients who died suggesting extensive death of progenitors in some patients [11]. This would explain the lack of difference between the two doses, and why probably even higher doses of leucovorin would be unlikely to improve recovery.

Similarly, for the same reason we feel it is unlikely that alternate antidotes like thymidine would improve mortality in these patients. Another antidote which has found use in the last decade is Glucarpidase. This works by hydrolyzing MTX in circulation and had found use in severe toxicity in context of HD-MTX with renal failure [12]. It is unlikely to find use in LD-MTX toxicity where levels are generally low. To our mind, better care of patients in intensive care units with aggressive treatment of infection and organ support is the most likely intervention to improve mortality in this scenario.

In the current study, serum albumin at presentation was found to be inversely associated with survival on multivariable analysis. Although previous studies have reported serum albumin to be a risk factor for MTX toxicity (for both HD and LD MTX) [13, 14], we did not find any previous report on its association with survival post-toxicity. A serum albumin higher than 3.1 g/dl had a modest predictive ability to predict survival. The lack of significant association with severity of leucopenia may be due to the selection of sick patients with severe leucopenia.

This study reiterates the most common reason of toxicity to be erroneous dosing (taking weekly dose either daily or alternate days). This is similar to previous studies starting from early studies in psoriasis where patient error was noted in 2/24 patients [15]. In RA, early on when MTX was introduced, MacKinnon and Wilkens noted four of their six patients with RA having MTX toxicity were confused about the dosing [4]. In a more contemporary series from India, toxicity in all patients was due to daily dosing due to poor understanding of the regimen [16]. The unique weekly-dosing of MTX, while convenient, is also associated with the specter of toxicity. Thus, it is pertinent that the physician takes time to emphasize that methotrexate should never be taken daily as it can be fatal. Furthermore, warnings on MTX tablets may help.

This study had limitations, most importantly of which was the small sample size. In addition, it was open label, although that is unlikely to influence the hard-end points like mortality or hematologic recovery. There was a significant difference in baseline albumin and cumulative methotrexate dose, due to a play of chance. The primary outcomes were adjusted for the former, however, in view of the cumulative dose being only available in 25 patients, it was not adjusted for the latter as that would have reduced the power of the study. We changed the primary outcome from time-to-hematological recovery to 30-day mortality (initially a secondary outcome) after the study had been initiated. This was done as it was thought to be more robust endpoint with more relevance considering the high mortality associated with the condition.

To conclude, this study did not find any significant difference in mortality between two doses of leucovorin 15 and 25 mg IV q6h in low-dose MTX associated severe toxicity. This suggests that a dose of 15 mg every 6 h remains appropriate for these patients. Whether a lower dose might suffice has not been studied yet, and would warrant future studies to find appropriate doses/ dosing schedule for leucovorin rescue.

## Conclusion

This study did not find any significant difference in mortality or hematological recovery between 15 (standard) or 25 mg IV q6h (higher) leucovorin in severe toxicity associated with low-dose MTX. As expected, most patients had either rheumatoid arthritis or psoriasis who had inadvertently taken methotrexate daily instead of weekly. This condition was found to have high mortality (in both groups), explained in part by the inclusion of sick patients (severe cytopenia) at baseline.

This is the first study to the best of our knowledge which has compared two different doses of leucovorin in low-dose MTX toxicity. This study suggests that there is no benefit of a higher dose over the standard dose of 15 mg leucovorin every 6 h. Based on this study no comments can be made of the adequacy of lower doses than 15 mg every 6 h. The high mortality in this study emphasizes the need for further research of other therapies and physicians should make sure they emphasize its unique dosing while prescribing methotrexate to patients and the consequences of inadvertent daily intake.

## Supplementary Information

**Additional file 1:** **Table S1.** Causes of death in the 17 patients who died in the first 30-days after randomization. **Table S2.** Receiver-Operating-Curve Analysis for serum albumin to predict survival. Area-under-curve=0.75, *p*=0.01. **Figure Supplementary 1.** Kaplan-meier survival curves showing time-to-event curves of oral ulcer recovery in the two groups. (log-rank test *p* =0.40, median estimate to mucositis recovery in days was 4, 5 days). **Figure Supplementary 2.** Kaplan-Meier survival curves showing time-to-event curves of WBC recovery (≥4 x 10^9/L) in the two groups. (log-rank test *p *=0.07, median estimate to WBC recovery in days was 4, 6 days). **Figure Supplementary 3.** Kaplan-meier survival curves showing time-to-event curves of partial platelet recovery (≥50 x 10^9/L) in the two groups.(log-rank test *p* =0.86, median estimate in days to platelet recovery in leucovorin 15 and 25 mg groups was 4, 4 days). **Figure Supplementary 4.** Kaplan-meier survival curves showing time-to-event curves of both WBC (≥4 x 10^9/L) and platelet recovery (≥50 x 10^9/L) in the two groups. (Log-rank test *p*=0.50, Median estimate in days in leucovorin 15 and 25 mg groups to both EBC and partial platelet recovery was 5, 6 days). **Figure S5.** Methotrexate polyglutamate 1-3 levels determined by HPLC.

## Data Availability

Data generated and analyzed in this study will be provided for reasonable requests; please send an email to the corresponding author.

## Abbreviations

95% CI: 95% Confidence interval
μM: Micro molar
AST: Aspartate aminotransferase
ALT: Alanine aminotransferase
CI: Confidence interval
CTRI: Clinical trials registry of India
DM: Diabetes mellitus
G-CSSF: Granulocyte colony stimulating factor
HT: Hypertension
IQR: Interquartile range (25–75 percentile)
IV: Intravenous
MTX: Methotrexate
q6h: Every 6 h
RA: Rheumatoid arthritis
SD: Standard deviation
WBC: White blood count
WBC nadir: Lowest white blood count
