# Supplementary material for: Comparison of two doses of leucovorin in severe low-dose methotrexate toxicity – a randomized controlled trial
Source: Arthritis Res Ther. 2023 May 19;25:82. doi: 10.1186/s13075-023-03054-2 (PMC10197821; doi:10.1186/s13075-023-03054-2)
Supplement: Supplementary file 1 — Additional file 1: Table S1. Causes of death in the 17 patients who died in the first 30-days after randomization. Table S2. Receiver-Operating-Curve Analysis for serum albumin to predict survival. Area-under-curve=0.75, p=0.01. Figure Supplementary 1. Kaplan-meier survival curves showing time-to-event curves of oral ulcer recovery in the two groups. (log-rank test p =0.40, median estimate to mucositis recovery in days was 4, 5 days). Figure Supplementary 2. Kaplan-Meier survival curves showing time-to-event curves of WBC recovery (≥4 x 10^9/L) in the two groups. (log-rank test p =0.07, median estimate to WBC recovery in days was 4, 6 days). Figure Supplementary 3. Kaplan-meier survival curves showing time-to-event curves of partial platelet recovery (≥50 x 10^9/L) in the two groups.(log-rank test p =0.86, median estimate in days to platelet recovery in leucovorin 15 and 25 mg groups was 4, 4 days). Figure Supplementary 4. Kaplan-meier survival curves showing time-to-event curves of both WBC (≥4 x 10^9/L) and platelet recovery (≥50 x 10^9/L) in the two groups. (Log-rank test p=0.50, Median estimate in days in leucovorin 15 and 25 mg groups to both EBC and partial platelet recovery was 5, 6 days). Figure S5. Methotrexate polyglutamate 1-3 levels determined by HPLC. [file 13075_2023_3054_MOESM1_ESM.docx]

**Supplementary Data**

**Comparison of Two Doses of Leucovorin in Severe Low-dose Methotrexate toxicity –A Randomized Controlled Trial**

Authors: Mudit Bhargava^1*^, Chirag Rajkumar Kopp^1*^, Shankar Naidu^1^, Deba Prasad Dhibar^2^, Atul Saroch^2^, Alka Khadwal^3^, Tarun Narang^4^, Siddharth Jain^1^, Aastha Khullar^1^, Bidyalaxmi Leishangthem^1^, Aman Sharma^1^, Susheel Kumar^1^, Shefali Sharma^1^, Sanjay Jain^1^, Varun Dhir^1^

Affiliations:

^1^Division of Rheumatology, Department of Internal Medicine, Postgraduate Institute of Medical Education and Research, Chandigarh 160012, India

^2^ Division of Emergency Medicine, Department of Internal Medicine Postgraduate Institute of Medical Education and Research, Chandigarh 160012, India

^3^Department of Clinical Hematology and Medical Oncology, Postgraduate Institute of Medical Education and Research, Chandigarh 160012, India

^4^Department of Dermatology, Postgraduate Institute of Medical Education and Research, Chandigarh 160012, India

Corresponding Author: Dr Varun Dhir, Professor, ^1^Division of Rheumatology, Department of Internal Medicine, Postgraduate Institute of Medical Education and Research, Chandigarh 160012, India

Email [varundhir@gmail.com](mailto:varundhir@gmail.com) Phone +91-8872229998

*Mudit Bhargava and Chirag Rajkumar Kopp contributed equally

| Tables | **Page number** |
| --- | --- |
| 1. Causes of Death | 1 |
| 1. ROC analysis for serum albumin for survival | 2 |
|  |  |
| Figures |  |
| 1. Kaplan-Meier survival curve for mucositis recovery | 3 |
| 1. Kaplan-Meier survival curve for WBC recovery (≥4 x10^9/L) | 4 |
| 1. Kaplan-Meier survival curve for partial platelet recovery (≥50 x 10^9/L) | 5 |
| 1. Kaplan-Meier survival curve for both WBC and partial platelet recovery (≥4 x 10^9/L and ≥50 x 10^9/L) | 6 |
| 1. Figure of RBC methotrexate polyglutamate-3 levels done by HPLC | 7 |
| Patient pictures | 8-10 |

Table S1: Causes of death in the 17 patients who died in the first 30-days after randomization

| **SNo.** | **Age** | **Gender** | **Leuco-**  **vorin dose** | **Disease** | **Cause of death** | **day of death** | **Hb** | **WBC 10^9/L** | **platelet X 10^9/L** |
| --- | --- | --- | --- | --- | --- | --- | --- | --- | --- |
| 1 | 54 | Female | 15mg | RA | sudden cardiac death | 10 | 6.4 | 3.4 | 110 |
| 2 | 50 | Male | 25mg | Psoriasis | septic shock, pneumonia, rising bilirubin (8.1mg/dl) | 5 | 9.1 | 1.3 | 6 |
| 3 | 52 | Female | 25mg | RA | septic shock, metabolic acidosis* | 9^1^ | 4.5 | 0.8 | 52 |
| 4 | 60 | Female | 25mg | RA | Shock, UGI bleed | 2 | 5.9 | 0.5 | 2 |
| 5 | 64 | Male | 15mg | RA | Shock, respiratory failure * | 7^2^ | 8.4 | 1.7 | 32 |
| 6 | 50 | Male | 25mg | RA | septic shock, AKI* | 6^3^ | 7 | 0.5 | 4 |
| 7 | 60 | Female | 25mg | RA | septic shock | 4 | 5.6 | 0.9 | 6 |
| 8 | 29 | Female | 15mg | Hydatiform Mole | COVID respiratory failure | 2 | 10.8 | 0.1 | 45 |
| 9 | 52 | Female | 15mg | RA | septic shock, pneumonia | 2 | 7.3 | 2.1 | 7 |
| 10 | 45 | Female | 25mg | Scleroderma | SRC, refractory acidosis, Septic shock | 18 | 7.7 | 28.7 | 52 |
| 11 | 48 | Female | 15mg | RA | AKI, respiratory failure (intubated), septic shock | 1 | 10.2 | 0.2 | 4 |
| 12 | 65 | Female | 15mg | RA | Unclear^#^ | 20^4^ | 9.3 | 12.9 | 126 |
| 13 | 72 | Male | 25mg | RA | Respiratory failure (intubated), septic shock | 1 | 7.9 | 0.6 | 7 |
| 14 | 56 | Male | 25mg | RA | septic shock, (intubated) | 11 | 7.1 | 20.5 | 52 |
| 15 | 66 | Male | 25mg | Psoriasis | septic shock | 2 | 8.7 | 0.4 | 3 |
| 16 | 29 | Male | 15mg | Unclear, CKD + AKI | sudden cardiac death | 3 | 11.7 | 0.2 | 5 |
| 17 | 61 | Female | 15mg | RA | Pneumonia, sudden cardiac death | 2 | 6.6 | 0.7 | 84 |

*Died after shifting to another medical facility/home-care # Died after discharge

^1^Remained in hospital (Leucovorin given) till day 7, ^2^Remained in hospital (Leucovorin given) till day 6, ^3^Remained in hospital (Leucovorin given) till day 4, ^4^Remained in hospital till day 19

Table S2 Receiver-Operating-Curve Analysis for serum albumin to predict survival. Area-under-curve=0.75, p=0.01


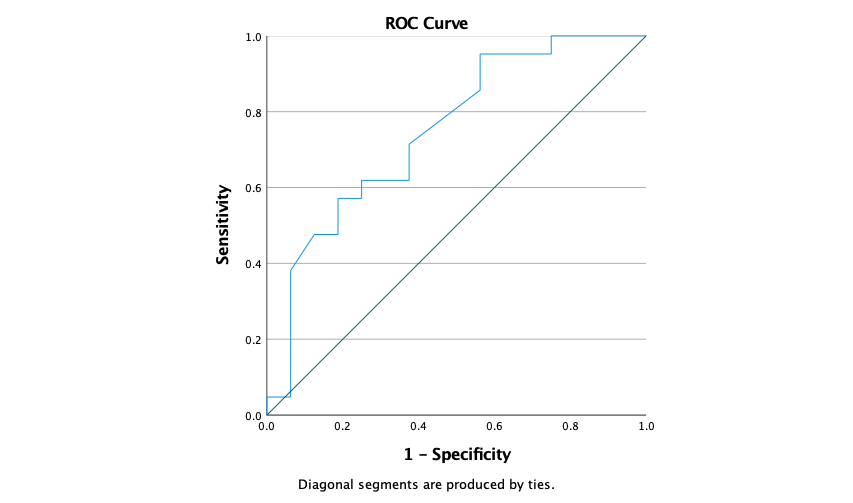


| Serum albumin  ≥ | sensitivity | 1-specificity | specificity | Add sensitivity and specificity |
| --- | --- | --- | --- | --- |
| 0.7 | 1 | 1 | 0 | 1 |
| 1.75 | 1 | 0.938 | 0.062 | 1.062 |
| 1.825 | 1 | 0.875 | 0.125 | 1.125 |
| 1.875 | 1 | 0.813 | 0.187 | 1.187 |
| 1.985 | 1 | 0.75 | 0.25 | 1.25 |
| 2.12 | 0.952 | 0.75 | 0.25 | 1.202 |
| 2.175 | 0.952 | 0.688 | 0.312 | 1.264 |
| 2.185 | 0.952 | 0.563 | 0.437 | 1.389 |
| 2.245 | 0.905 | 0.563 | 0.437 | 1.342 |
| 2.35 | 0.857 | 0.563 | 0.437 | 1.294 |
| 2.45 | 0.81 | 0.5 | 0.5 | 1.31 |
| 2.55 | 0.714 | 0.375 | 0.625 | 1.339 |
| 2.62 | 0.667 | 0.375 | 0.625 | 1.292 |
| 2.645 | 0.619 | 0.375 | 0.625 | 1.244 |
| 2.69 | 0.619 | 0.313 | 0.687 | 1.306 |
| 2.84 | 0.619 | 0.25 | 0.75 | 1.369 |
| 2.975 | 0.571 | 0.25 | 0.75 | 1.321 |
| 3.05 | 0.571 | 0.188 | 0.812 | 1.383* |
| 3.115 | 0.476 | 0.188 | 0.812 | 1.288 |
| 3.165 | 0.476 | 0.125 | 0.875 | 1.351 |
| 3.245 | 0.381 | 0.063 | 0.937 | 1.318 |
| 3.345 | 0.333 | 0.063 | 0.937 | 1.27 |
| 3.425 | 0.286 | 0.063 | 0.937 | 1.223 |
| 3.51 | 0.238 | 0.063 | 0.937 | 1.175 |
| 3.635 | 0.19 | 0.063 | 0.937 | 1.127 |
| 3.75 | 0.095 | 0.063 | 0.937 | 1.032 |
| 3.85 | 0.048 | 0.063 | 0.937 | 0.985 |
| 3.955 | 0.048 | 0 | 1 | 1.048 |
| 5.01 | 0 | 0 | 1 | 1 |

*Highest sum of sensitivity and specificity

Figure Supplementary 1: Kaplan-meier survival curves showing time-to-event curves of oral ulcer recovery in the two groups.

(log-rank test p =0.40, median estimate to mucositis recovery in days was 4, 5 days)


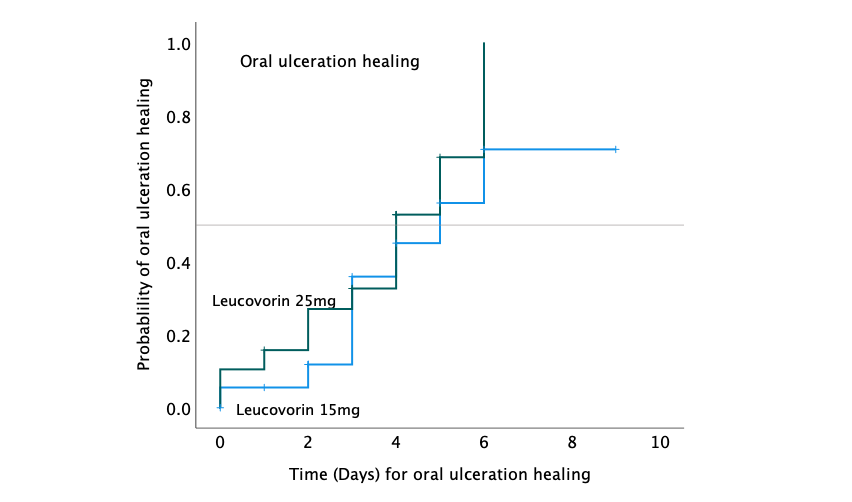


Figure Supplementary 2: Kaplan-Meier survival curves showing time-to-event curves of WBC recovery (≥4 x 10^9/L) in the two groups. (log-rank test p =0.07, median estimate to WBC recovery in days was 4, 6 days)


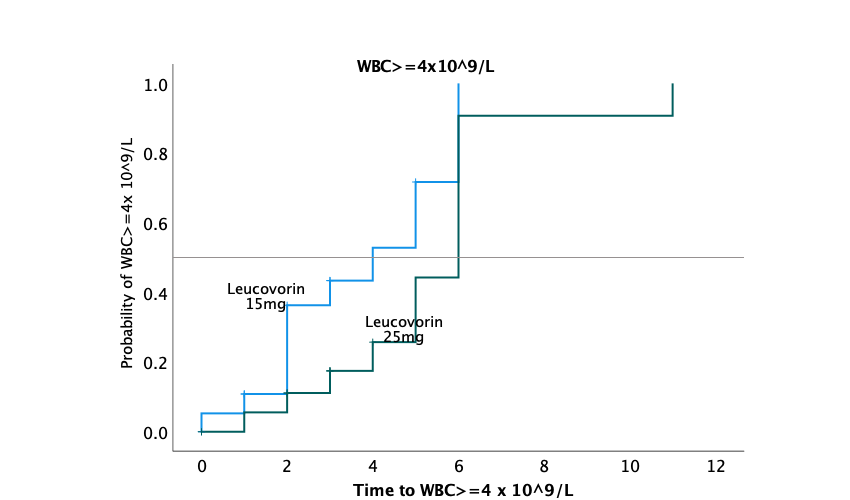


Figure Supplementary 3: Kaplan-meier survival curves showing time-to-event curves of partial platelet recovery (≥50 x 10^9/L) in the two groups.(log-rank test p =0.86, median estimate in days to platelet recovery in leucovorin 15 and 25 mg groups was 4, 4 days)


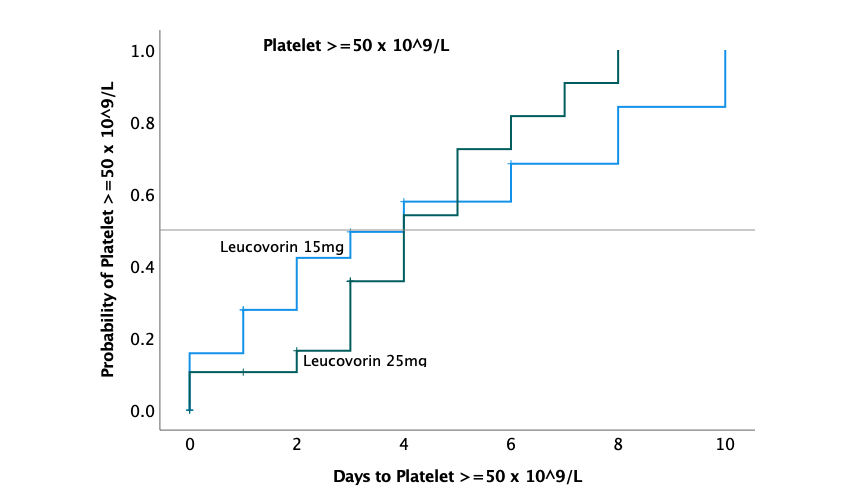


Figure Supplementary 4: Kaplan-meier survival curves showing time-to-event curves of both WBC (≥4 x 10^9/L) and platelet recovery (≥50 x 10^9/L) in the two groups.

(Log-rank test p=0.50, Median estimate in days in leucovorin 15 and 25 mg groups to both EBC and partial platelet recovery was 5, 6 days)


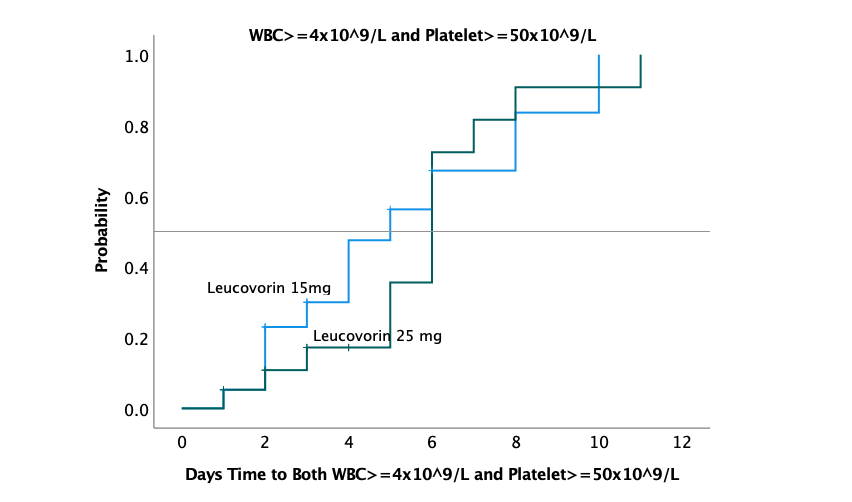


Figure S5. Methotrexate polyglutamate 1-3 levels determined by HPLC.

|  | PG1 (Nmol/L) | PG2 (Nmol/L) | PG3 (Nmol/L) | Total  (Nmol/L) |
| --- | --- | --- | --- | --- |
| Patient 1 | 26.68 | 40.08 | 66.91 | 133.67 |
| Patient 2 | 25.32 | 28.25 | 30.57 | 84.14 |
| Patient 3 | 40.59 | 33.92 | 42.39 | 116.9 |
| Patient 4 | 141.09 | 90.50 | 41.30 | 272.89 |
| Patient 5 | 20.32 | 20.83 | 22.43 | 63.58 |
| Patient 6 | 44.95 | 45.00 | 63.87 | 153.82 |
| Patient 7 | 31.91 | 23.25 | 30.00 | 85.16 |
| Patient 8 | 42.34 | 11.64 | 16.42 | 70.4 |
| Patient 9 | 13.47 | 2.31 | 2.53 | 18.31 |
| Patient 10 | 45.02 | 21.64 | 47.49 | 114.15 |
| Patient 11 | 8.98 | - | 4.39 | 13.37 |
| Patient 12 | 17.43 | 4.15 | 2.53 | 24.11 |

Water with PG1 to PG 5 standards (PG3 is at 15.230 minutes)

RBC lysate of a patient with MTX toxicity showing PG1 peak at 20.8, PG2 at 17.7 and PG3 peak at 15.4

A reverse-phase high-performance liquid chromatography (HPLC) was used for chromatographic separation of MTX polyglutamates (PG) according to the procedure described by Dervieux et al. The Agilent 1290 Infinity LC chromatographic system consisted of (Agilent Technologies, Santa Clara, California, USA ) with a binary pump with integrated degasser, a system controller, auto-injector, fluorometric detector, diode array detector, thermostat column compartment and a sample cooler kept at 4 degree Celsius. A Thermo C18 column (Thermo Fisher Scientific, Sunnyvale, CA) of 250 x 4.6 mm (inner diameter) 5 um particle size were used with for metabolite separation. It was followed by post-column photooxidation carried out using a photochemical reactor unit (PHRED Photochemical detector; Aura Industries, NY) equipped with a 254-nm low-pressure mercury ultraviolet lamp using ultraviolet irradiation in the presence of hydrogen peroxide. It was connected online of output of the analytical column and as input of the fluorometric detector. Mobile phase A consisted of 10 mmol/L ammonium acetate containing 2 mL/L hydrogen peroxide and mobile phase B consisted of HPLC grade acetonitrile. CDS Chemstation Edition software (Agilent Technologies, Santa Clara, California, USA) was used to acquire and analyse chromatograms. Patients’ blood samples were collected in EDTA vials, centrifuged, and supernatant (plasma) was removed. Aliquots of 500 uL of packed RBCs were stored at -80 degree Celsius after washing twice with cold phosphate-buffered saline. Stored samples of packed RBC were removed from the -80 degree celsius freezer and thawed repeatedly. Volume of 750 uL of water and 125 uL of 700 mL/L perchloric acid (for deproteinization) were added to 500 uL of thawed RBC lysate. Then vortexed and centrifuged at 12000 rpm for 10 mins. A volume of 80 uL of the supernatant was injected into the HPLC system using an autoinjector. Initially, a 20-minute linear gradient from 0% to 13% acetonitrile (mobile phase B) at a flow rate of 1 mL/min was done to separate MTX-glu1 to MTX-glu5. The mobile phase was returned to 100% mobile phase A after 20 minutes and re-equilibrated for 10 minutes. The photolytic product of MTX-glu_n_ was measured at an excitation wavelength of 274 nm and emission wavelength of 470 nm. Peaks were identified by overlaying the peaks for both patient samples and polyglutamate standards in RBC lysate as well as in water (purchased from Schircks Laboratories, Jona, Switzerland). Different concentrations of single polyglutamates (7.5, 15.6, 31.2, 62.5 and 125nM) standards were made by spiking control RBCs. Concentrations in patient samples were calculated from standard curves and linear regression equations. Due to overlap of MTX PG-4 and 5 with matrix peaks, these could not be reliably estimated.

**References**

Dervieux T, Orentas Lein D, Marcelletti J, et al. HPLC determination of erythrocyte methotrexate polyglutamates after low-dose methotrexate therapy in patients with rheumatoid arthritis. Clin Chem. 2003; 49:1632–1641.

Jain S, Dhir V, Aggarwal A, et al. Comparison of two dose escalation strategies of methotrexate in active rheumatoid arthritis: a multicentre, parallel group, randomised controlled trial. Ann Rheum Dis . 2021 Nov; 80(11):1376-1384.

Patient Picture 1


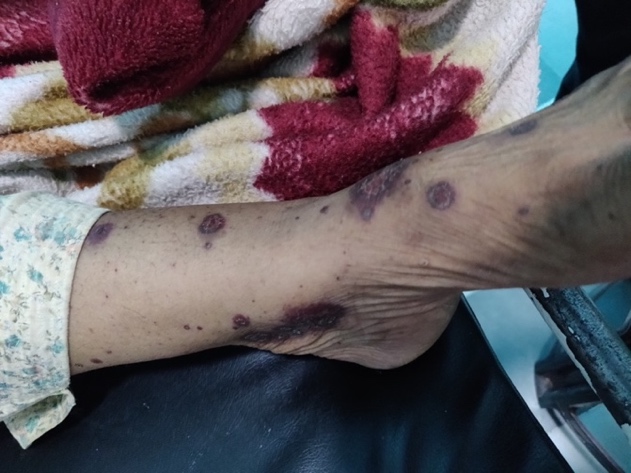

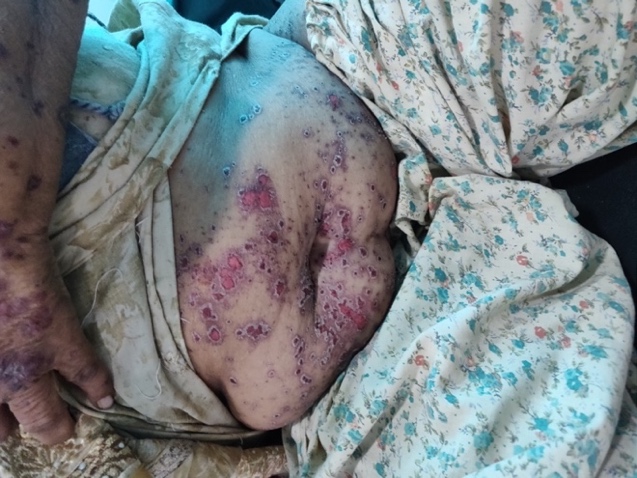

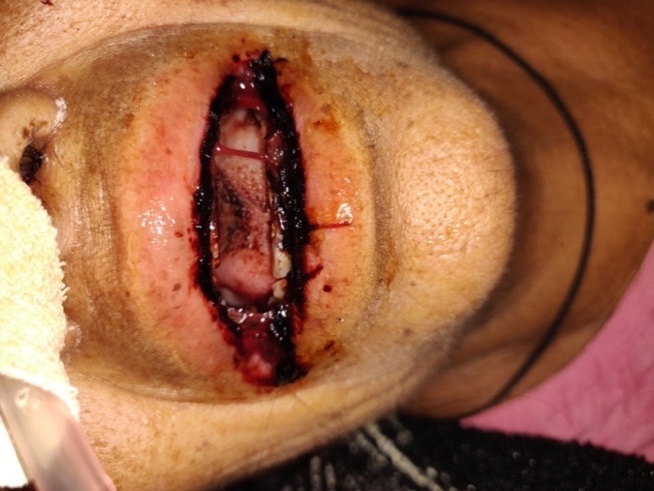

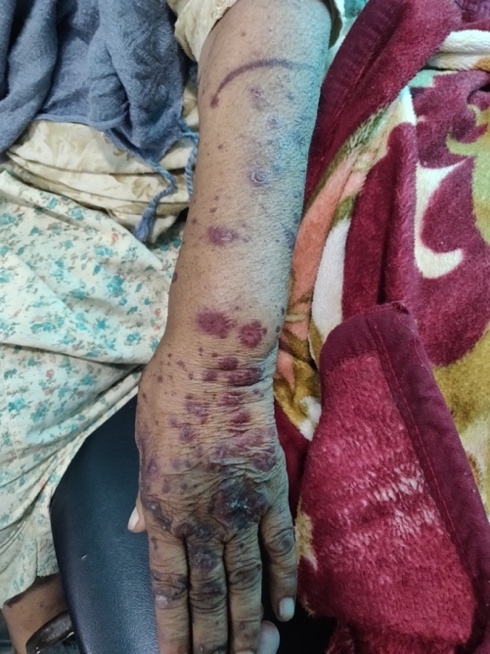
A B

C D

79/F Psoriasis with erroneous intake of MTX 22.5 mg daily for 7 days (A) oral mucositis with necrotic sludge on the lips (B, C, D) Ulceration of preexisting psoriatic plaques on forearm, abdomen, and leg

Patient Picture 2


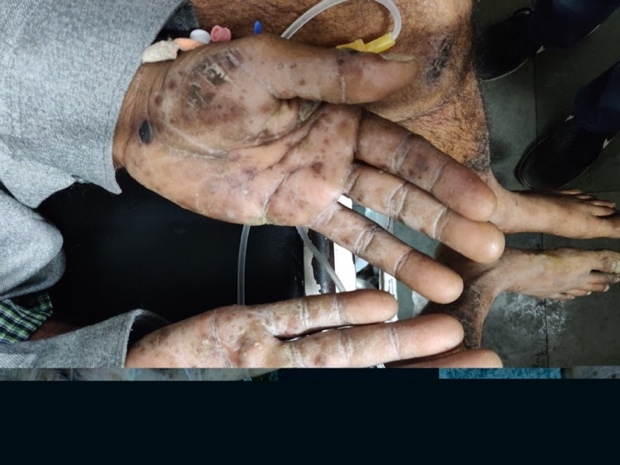

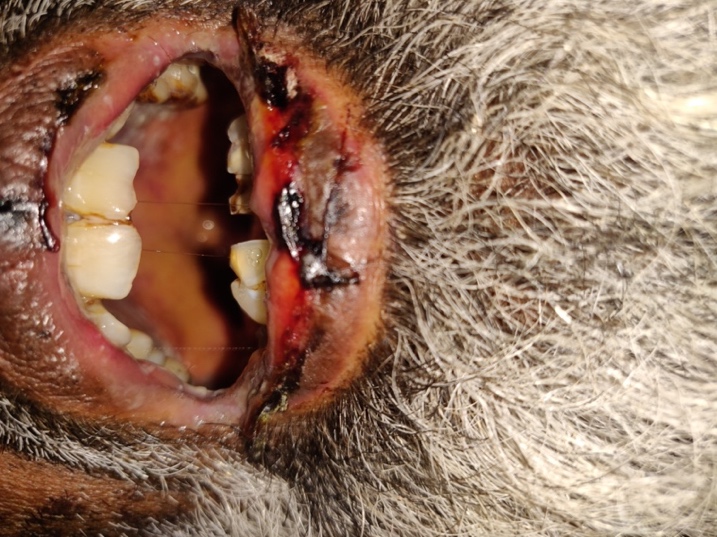
A B


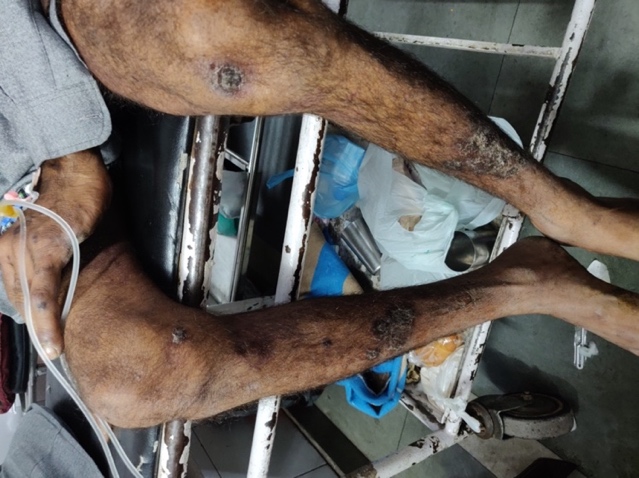

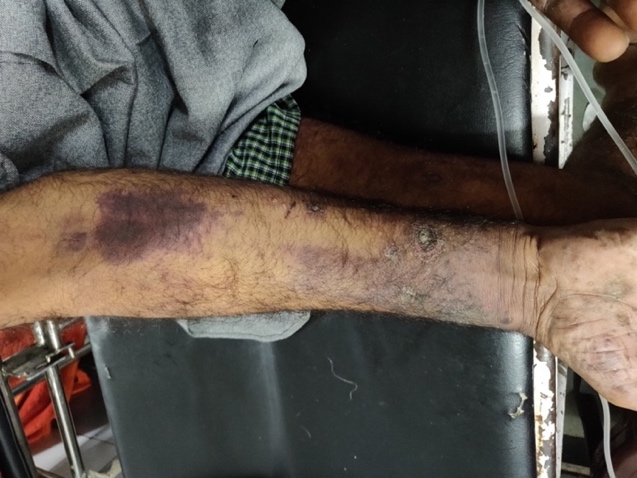


C D

50/m Patient with Psoriasis with Methotrexate 5 mg daily for 15 days. (A) Oral mucositis with necrotic areas on lips (B) Targetoid lesions on palms (C, D) Legs and forearm showing superficial erosions of psoriatic plaques. In addition, large purpura on the superior aspect of forearm


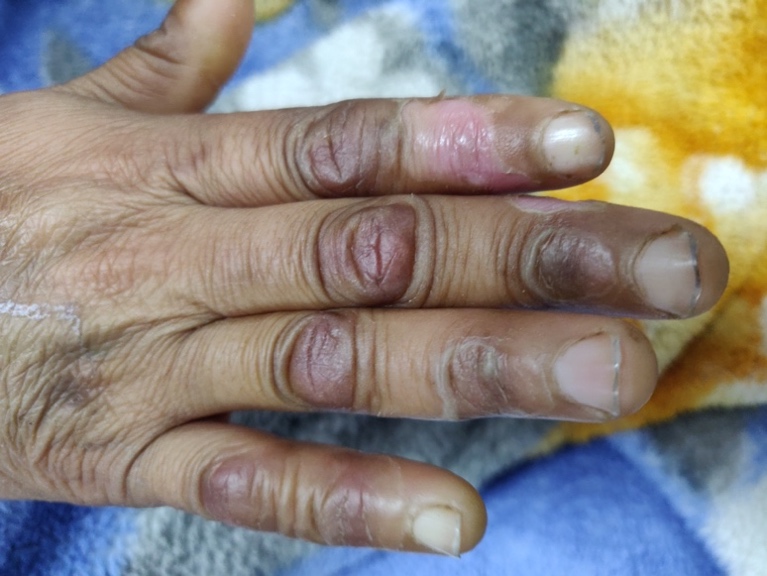


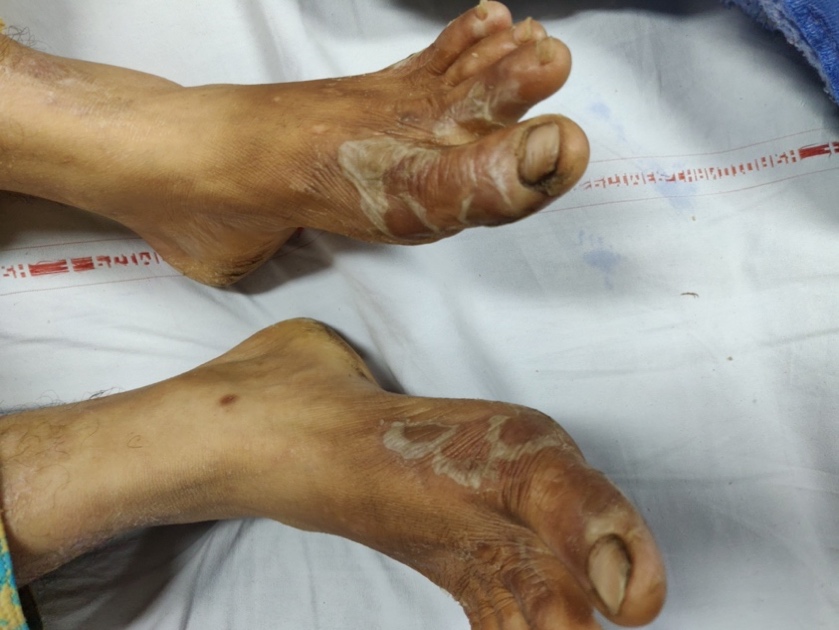
Patient Picture 3

A B

35/F with psoriasis overdose 67.5mg

1. Superficial erosions and bullae over dorsal surface of hands (B) Flaccid bullae on the feet

Patient Picture 4


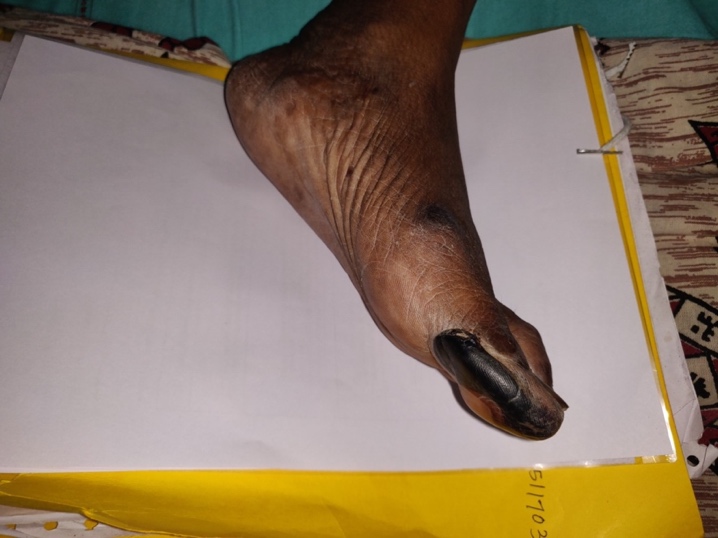

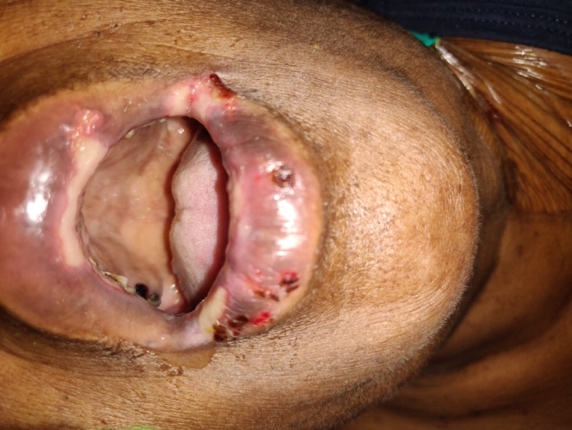


A B

40/f with RA with MTX overdose.7.5 mfg OD x 7 days

1. Oral mucositis with erosions on lips and edema (B) Large hemorrhagic bullae of 4x1 cms over the medial aspect of the foot
